# Non-neutral clonal selection and its potential role in mammalian germline stem cell dysfunction with advancing age

**DOI:** 10.3389/fcell.2022.942652

**Published:** 2022-08-23

**Authors:** Victor Stolzenbach, Dori C. Woods, Jonathan L. Tilly

**Affiliations:** Laboratory of Aging and Infertility Research, Department of Biology, Northeastern University, Boston, MA, United States

**Keywords:** germline stem cell, oogonial stem cell, spermatogonial stem cell, aging, ovary

## Abstract

The concept of natural selection, or "survival of the fittest", refers to an evolutionary process in nature whereby traits emerge in individuals of a population through random gene alterations that enable those individuals to better adapt to changing environmental conditions. This genetic variance allows certain members of the population to gain an advantage over others in the same population to survive and reproduce in greater numbers under new environmental pressures, with the perpetuation of those advantageous traits in future progeny. Here we present that the behavior of adult stem cells in a tissue over time can, in many respects, be viewed in the same manner as evolution, with each stem cell clone being representative of an individual within a population. As stem cells divide or are subjected to cumulative oxidative damage over the lifespan of the organism, random genetic alterations are introduced into each clone that create variance in the population. These changes may occur in parallel to, or in response to, aging-associated changes in microenvironmental cues perceived by the stem cell population. While many of these alterations will be neutral or silent in terms of affecting cell function, a small fraction of these changes will enable certain clones to respond differently to shifts in microenvironmental conditions that arise with advancing age. In some cases, the same advantageous genetic changes that support survival and expansion of certain clones over others in the population (viz. non-neutral competition) could be detrimental to the downstream function of the differentiated stem cell descendants. In the context of the germline, such a situation would be devastating to successful propagation of the species across generations. However, even within a single generation, the “evolution” of stem cell lineages in the body over time can manifest into aging-related organ dysfunction and failure, as well as lead to chronic inflammation, hyperplasia, and cancer. Increased research efforts to evaluate stem cells within a population as individual entities will improve our understanding of how organisms age and how certain diseases develop, which in turn may open new opportunities for clinical detection and management of diverse pathologies.

## Introduction

Resident stem cells have been identified in most organs of the body, providing a means for tissue growth and for the replacement of damaged or aged cells over time (Morrison and Spradling, 2008). Because of their central importance to the development, homeostatic maintenance, and function of adult tissues, tremendous research efforts are focused on identifying the source (intrinsic vs. extrinsic to the tissue), characteristic features (gene expression profiles), regulation (self-renewal vs. differentiation), developmental potential (lineage-restricted vs. multi-potent), and clinical utility of both somatic and germline stem cells. As this work has progressed, an intriguing concept regarding adult stem cell behavior has emerged: the response of individual stem cells within a population to the same stimulus, whether it be a morphogen, a metabolic modulator or even a subtle change in biomechanical strain (Ito and Ito, 2016; Vining and Mooney, 2017; Camacho-Aguilar and Warmflash, 2020; Ly et al., 2020; MacDonald et al., 2021), can vary widely, with some cells committing to differentiation while others remain in a quiescent state (He et al., 2009). In short, stem cells within the same population are not uniformly the same, and the degree of heterogeneity across stem cells of a given population changes as organisms age.

It has been established that pools of undifferentiated stem cells persist in adults throughout life (Morrison and Spradling, 2008; Gurusamy et al., 2018; Mohammad et al., 2019), and it is highly likely that these cells must constantly adapt to changes in their microenvironments or risk elimination as nearby stem cells outcompete them for limited space and resources (Stine and Matunis, 2013). This niche dependence, in which small fractions of stem cells reside in microenvironments tailored to allow them to exist in an undifferentiated or semi-differentiated state, is observed in a variety of adult stem cell populations, including those of the hematopoietic, germline, gastrointestinal and epidermal lineages (Morrison and Spradling, 2008; Gonzales and Fuchs, 2017; Fayomi and Orwig, 2018; Gehart and Clevers, 2019; Martin et al., 2019). As the body ages, both cell-intrinsic and cell-extrinsic changes lead to disruptions in the capacity of stem cells to maintain the population through self-renewal as well as in the ability of the stem cell progeny to differentiate and perform their lineage-specific function(s) in the tissue supported by that population (Jung and Brack, 2014; Goodell and Rando, 2015; García-Prat and Muñoz-Cánoves, 2017; de Haan and Lazare, 2018; Keyes and Fuchs, 2018; Sameri et al., 2020). Although stem cell numbers in some tissues decline with age, this is not a general observation across all stem cell lineages. In fact, current evidence discussed below points to a disruption of normal stem cell function, and not a numerical loss of stem cells per se, as being the most critical contributor to aging-related deterioration of organ function.

Changes in niche composition and extrinsic support of stem cells occur with age (Singh, 2012; Pinho and Frenette, 2019), and these events are unquestionably involved in facilitating, or even driving, alterations in gene expression, self-renewal capacity, and the differentiation potential of various types of stem cells (Niikura et al., 2009; Ji et al., 2017; Latchney and Calvi, 2017; Ge et al., 2020; Ho and Méndez-Ferrer, 2020; Pentinmikko and Katajisto, 2020). However, there is a far more complicated view of this basic model, which entails changes in microenvironmental conditions occurring in parallel to, and in some cases independent of, genetic changes among individual stem cells within a population. This latter process, which we refer to here as clonal heterogeneity, arises in large part either because of random mutations introduced during DNA replication associated with each stem cell division or through cumulative oxidative damage in individual stem cells over time. While most of these genetic events will be neutral or silent–in that, the normal patterns of gene expression in, or the function of, the stem cell harboring that alteration remain unaffected, some of these events can lead to changes in gene expression and, consequently, the ability of a given stem cell within the population to continue to thrive or function in the face of shifting niche conditions. In some stem cell models, environmental pressures can therefore drive selection of individual stem cells, or clones, within a population that have acquired, through random chance, advantageous gene alterations which facilitate “niche independence”. As support from the niche continues to erode with advancing age, other clones that are still reliant on normal niche function to survive and self-renew are lost. These members of the population are then gradually replaced with more and more of the mutant clones in the face of continued pressure from a suboptimal niche that permits survival of only the “fittest” cells through a process referred to as non-neutral competition.

**FIGURE 1:**
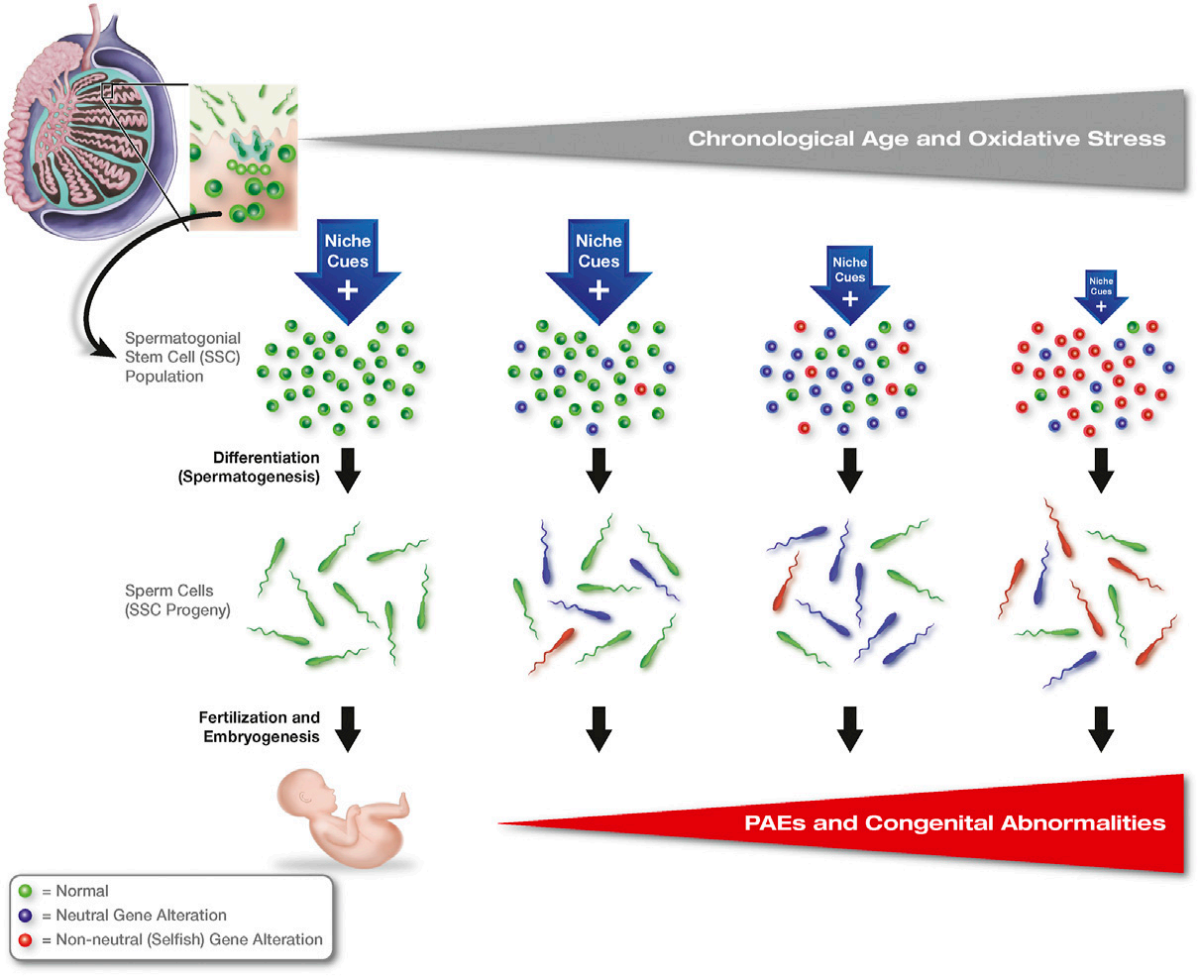
Clonal heterogeneity and non-neutral selection in adult stem cell populations: selfish selection in SSCs. Genetic alterations occur randomly within individual SSCs with age, most of which are neutral or silent (blue-shaded cells); however, some mutations lead to disruptions in FGFR/RAS/MAPK signaling in affected clones (selfish mutations, red-shaded cells), which enable survival and growth of these stem cells independent of niche-derived signals normally needed by SSCs to function. As support from the niche deteriorates with age, SSC clones with neutral mutations that are still reliant on signals from the niche are lost and replaced with SSCs harboring genetic alterations that convey niche independence, leading to an expansion of these clones across the population as other clones lacking advantageous mutations progressively die off. While these latter mutations are advantageous to the selected stem cell clones, spermatozoa produced by these SSCs carry the same mutations into eggs at fertilization, leading to a spectrum of developmental abnormalities, or paternal age effects (PAEs), associated with aberrant FGFR/RAS/MAPK signaling during embryogenesis.

In some respects, this model would be attractive as a mechanism for maintaining stem cell numbers in tissues with age, even as niche support of cells in that population deteriorates. However, this form of non-neutral clonal selection can have devastating downstream consequences if a gene alteration that allows for certain stem cells in the population to lose their reliance on the niche is one that is not conducive for proper function of the descendants of those stem cells once differentiation has occurred. As will be discussed below, the occurrence of such a process has been demonstrated in male germline or spermatogonial stem cells (SSCs) of the adult human testis (see Section 2. Selfish Selection in Spermatogonial Stem Cells). In this model, an aging-related expansion of SSC clones harboring advantageous mutations that convey niche independence comes at a significant cost. Namely, the same mutations transmitted through the progeny of mutant SSCs to embryos conceived by those sperm cells disrupt normal fetal development, leading to a spectrum of offspring abnormalities (Thacker, 2004; Goriely and Wilkie, 2012). Accordingly, non-neutral clonal selection in SSCs is often referred to as “selfish” selection since the mutations that provide a growth or survival benefit to certain stem cells in the face of changing niche conditions are detrimental to the downstream purpose of those cells in the organism (Figure 1). Interestingly, a somewhat different form of non-neutral clonal selection occurs in hematopoietic stem cells (HSCs) with age, and this process has been associated with numerous blood cell pathologies, including immunological compromise, anemia, and cancer (Bowman et al., 2018; Shlush, 2018; Jaiswal and Ebert, 2019), as well as several non-hematologic diseases (Jaiswal, 2020). However, in this model the selection and expansion of HSC clones with age is far more complicated to evaluate because, unlike SSCs which are unipotent *in vivo*, HSCs are multipotent progenitors for the production an array of differentiated cells that comprise both the myeloid and lymphoid lineages. Moreover, immune cells derived from HSCs are central to inflammatory responses, which indirectly influence many other tissues outside of the blood cell system. Hence, changes in HSC function with age are multifactorial and are not the end-result of a single type of initiating event or common process, as is the case for clonal selection in SSCs and spermatogenesis.

**FIGURE 2:**
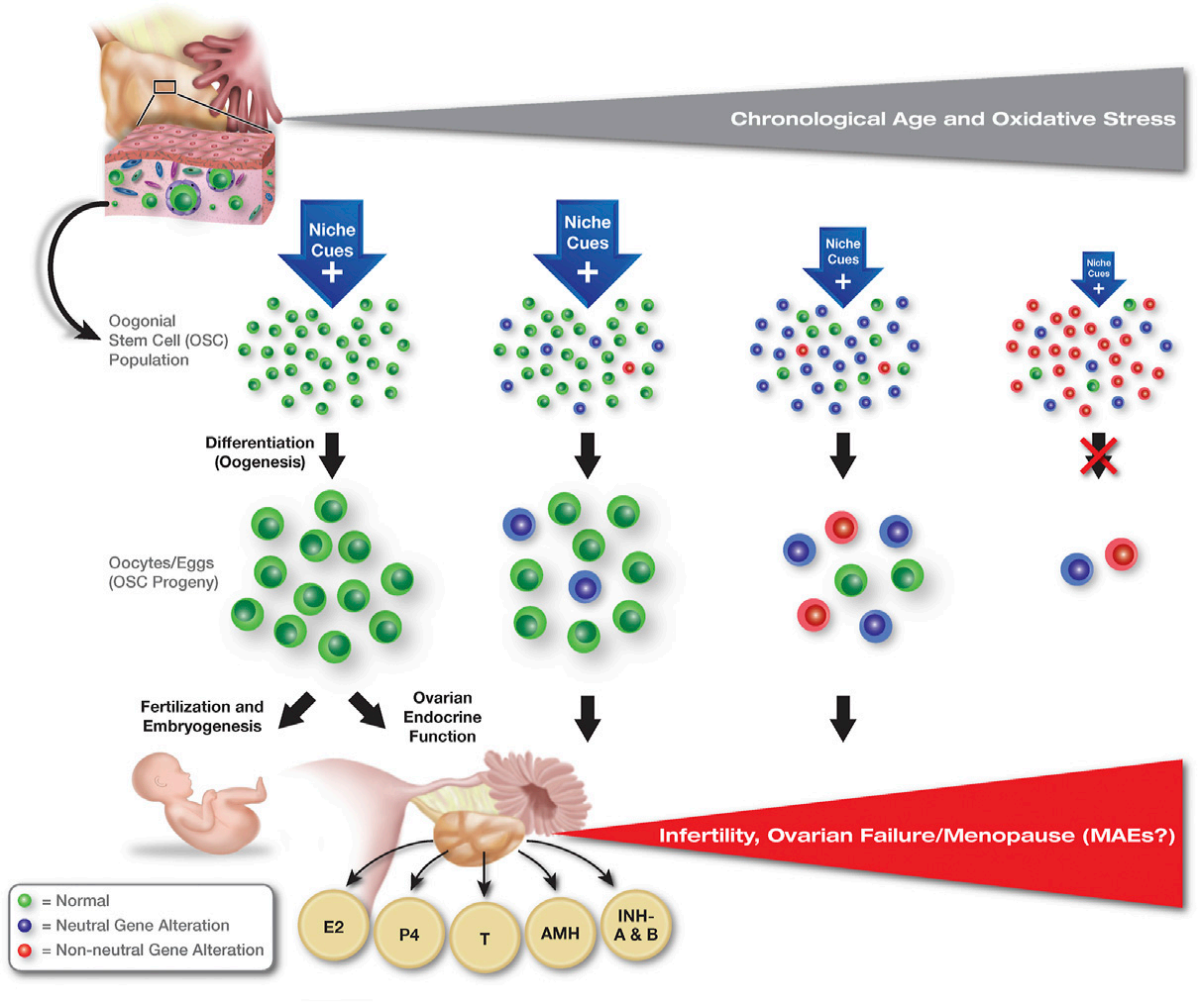
Hypothesized role of clonal heterogeneity and non-neutral selection in the progressive quiescence of OSCs with age. Contrasting the continuation of spermatogenesis in males at advanced chronological ages, even in the face of selfish SSC selection (see Figure 1), oogenesis in females shuts down with age. Accordingly, oocyte loss through follicle atresia is no longer partially offset by the formation of new oocyte-containing follicles, leading to exhaustion of the oocyte reserve and ovarian failure (see Wang et al., 2017 for details). Oogonial stem cells are known to persist in ovaries into advanced ages, but the cells lose the capacity to differentiate into new oocytes *in vivo*. These findings suggest that OSCs become quiescent with age, although the cues that drive this, and the mechanisms underlying the progressive impairment in differentiation potential, are currently unknown. Abbreviations: E2, estrogen; P4, progesterone; T, testosterone; AMH, anti-Müllerian hormone; INH-A and B, inhibin-A and -B.

These findings raise several important questions for the field of stem cell biology to consider. First, are clonal heterogeneity and non-neutral clonal selection with age fundamental principles that apply to all types of adult stem cell populations or only to a select few? Second, does gene mutation-based clonal selection in stem cell populations always involve alterations that ultimately disrupt the downstream function of the stem cell progeny, such that aging-associated tissue pathologies can be traced back to a select few mutant clones? Third, how many forms of non-neutral clonal selection are there, and are the consequences of each the same or different? And finally, to what extent does clonal heterogeneity and non-neutral clonal selection unknowingly affect our understanding of stem cell biology based on results obtained from stem cells maintained in cultures that are, without question, “suboptimal” compared to the *in-vivo* microenvironments that the stem cells normally reside in. In other words, does *in-vitro* culture inadvertently drive the selection of only those stem cell clones that, by virtue of random genetic differences, become best suited to grow under non-physiological conditions *ex-vivo*? If this is the case, are observations with stem cells maintained in culture reflective of the stem cell population as a whole or of only a small fraction of the population that has gained a growth advantage due to clonal heterogeneity? To explore these questions in more detail, here we overview what is known of non-neutral clonal selection in SSCs and HSCs as two established models of this phenomenon in mammals. We then use this information as a preface for evaluating the possibility that some form of non-neutral clonal selection also occurs in female germline or oogonial stem cells (OSCs) during ovarian aging, and that such a process may contribute to a decline in female reproductive fitness over time (Figure 2). Finally, we highlight how a greater understanding of microenvironmental pressures, clonal heterogeneity, and non-neutral clonal selection in stem cell populations may lead to new opportunities for detection and management of congenital abnormalities, disease initiation, and aging-associated pathologies.

## Selfish selection in spermatogonial stem cells

Male germline stem cells comprise the most primitive form of spermatogonia in the testes. These adult stem cells, which exist within a niche microenvironment, are the source of all mature sperm cells, or spermatozoa, produced throughout life in males (Huckins, 1978; Brinster, 2007; Fayomi and Orwig, 2018; Kubota and Brinster, 2018; Kubota, 2019). Lifelong maintenance of spermatogenesis, even into advanced ages, is therefore dependent on SSCs and their continual self-renewal. Throughout much of adult life, the number of SSCs in the testis remains relatively stable due to asymmetric divisions, in which one of the 2 cells produced by each stem cell mitotic event will differentiate while the other cell remains undifferentiated and returns to a state of quiescence. Functionally, these cells are defined by their long-term ability to self-renew and to differentiate into mature gametes after transplantation into testes of infertile hosts (Brinster and Avarbock, 1994; Brinster and Zimmermann, 1994; Kanatsu-Shinohara et al., 2016; Kubota, 2019). Although males are generally believed to retain fertile potential throughout most of their adult lifespan, SSC-supported spermatogenesis still shows signs of dysfunction with advancing age. For example, one study utilizing transplantation-based approaches in mice demonstrated that the ability of donor SSCs to engraft in host testes and establish spermatogenesis declines significantly as the age of the male recipient increases (Ryu et al., 2006). These findings, coupled results from studies with mice which performed serial transplantation of genetically traceable SSCs every 3 months for 3 years, indicate that deterioration of the SSC niche with age is a major contributory factor to the loss of SSC function (Ryu et al., 2006).

However, evidence of intrinsic changes in SSCs with age also exists. As mentioned earlier, a particularly intriguing aspect of aging and SSCs involves a process that is now commonly referred to as selfish selection (Glaser et al., 2003; Goriely et al., 2003; Thacker, 2004; Goriely and Wilkie, 2012; Goriely et al., 2013; Kong et al., 2012; Maher et al., 2014; Maher et al., 2018). This phenomenon involves the occurrence of random gene mutations arising in SSCs during each cell division, most of which are silent in terms of cell function. However, some of these mutations confer a survival advantage to certain SSC clones, such that these specific cells within the population can survive, divide, and differentiate independent of the external or niche-derived cues normally needed by SSCs to function properly. As support from the SSC niche progressively deteriorates with age, SSC clones with advantageous mutations outcompete other SSCs lacking these mutations. The SSC population, as an entity, then gradually shifts to one dominated by these mutant SSCs through non-neutral competition, which results in a corresponding upward shift in the frequency of spermatozoa being generated that carry the same mutations (Goriely and Wilkie, 2012; Lim et al., 2012; Maher et al., 2014) (Figure 1). Among the mutations prominent in selfish SSC selection are those found in genes that encode proteins comprising the tyrosine kinase receptor/RAS/MAPK (mitogen-activated protein kinase) signaling pathway (Goriely et al., 2003; Maher et al., 2018). The most common SSC mutations result in constitutive (non-ligand dependent) activation of FGFR2 (fibroblast growth factor receptor 2) and SHP (Src homology region 2-containing protein tyrosine phosphatase 2), the latter of which is encoded by the *PTPN11* (protein tyrosine phosphatase non-receptor type 11) gene. Mechanistically, FGFR2 is a member of a family of receptors that has long been implicated in cancer development (Kuroda et al., 2020). Mutations in the *PTPN11* gene have been directly linked to a spectrum of pathologies, including childhood leukemia and Noonan syndrome, the latter of which is an autosomal dominant genetic disorder that is inherited in a strictly paternal manner (Tartaglia et al., 2002; Zhang et al., 2015a).

Broadly speaking, spermatogenic dysfunction with age is widely thought to be responsible for a phenomenon known as the paternal age effect (PAE) (Thacker, 2004). An underlying principle of the PAE is that offspring sired by older males will be at a higher risk for monogenetic birth defects and disorders that arise from advantageous gene mutations which occur in SSCs as this population of adult stem cells undergoes clonal selection during aging (Glaser et al., 2003; Goriely and Wilkie, 2012; Maher et al., 2014). These PAE-linked mutations result in a variety of clinical disorders in offspring, including Apert syndrome (malformation of skull, face, hands, and feet), Crouzon syndrome (craniofacial defects), Pfeiffer syndrome (malformation of skull, face, hands, and feet), achondroplasia (skeletal defects, short stature), and thanatophoric dysplasia (small ribcage, stunted limb development). While most PAEs appear to be associated with craniofacial or skeletal abnormalities, PAEs have also been linked to multiple endocrine neoplasia type 2 and type 2b (tumors in endocrine organs, including adrenals, thyroid glands, and parathyroid glands, as well as tumors of the eyes and mouth), schizophrenia, and autism.

As stated earlier, many of the identified mutations that result in PAEs target different components of the FGFR/RAS/MAPK signaling pathway. These same genes also regulate the self-renewal and differentiation of SSCs. For example, one of the ligands in the signaling cascade involving FGFR activation is GDNF (glial-derived neurotrophic factor), which is critical for maintaining the pool of SSCs throughout adult life (Meng et al., 2000). Additionally, activation of ERK (extracellular signal-regulated kinase) and AKT (AK strain transforming 1 kinase) signaling by FGFs has been identified as a key mechanism involved in promoting SSC proliferation as well as in the competitive selection of SSC clones within the testicular niche (Kitadate et al., 2019; Tian et al., 2019). Use of Markov chain models along with mutation assays of donor sperm have been used to confirm a direct connection between PAE-associated birth defects and SSC dysfunction that can be traced to sperm carrying a variety of FGFR/RAS/MAPK pathway mutations. In other words, the mutations which confer selective advantages to certain SSCs in their deteriorating niche come at a cost: the genes affected are the same ones that drive PAEs in offspring conceived with sperm carrying those mutations (Goriely and Wilkie, 2012; Whelan et al., 2016). The prognostic value of the Markov chain modeling approach is striking in that its use predicts incidence rates for Apert syndrome, Costello syndrome, and thanatophoric dysplasia that are extremely close to the actual observed values (Whelan et al., 2016). Taken together, these findings support a crucial role for selfish clonal selection in SSCs as a principal contributing factor to aging-associated testicular dysfunction and spermatogenic defects, as well as the prognostic value of screening spermatozoa prior to use in assisted human reproductive technologies for transmissible gene mutations that would be detrimental to offspring development (Breuss et al., 2022).

## Lineage skewing during clonal hematopoiesis

Hematopoietic stem cells, localized within specialized bone marrow niches (Baum et al., 1992; Morrison and Scadden, 2014), serve as the progenitors to billions, or even trillions, of blood cells that are produced and circulate throughout the body over the course of postnatal life (Seita and Weissman, 2010). The majority of cellular turnover in the body occurs in cells of the hematopoietic lineage (Sender and Milo, 2021), and as such HSCs are critical for maintaining a healthy population of differentiated blood cells throughout lifespan. In general, HSCs are categorized as long-term (LT) or short-term (ST) reconstituting cells, with LT-HSCs being the most primitive and capable of self-renewal. In turn, ST-HSCs arise from LT-HSCs as a slightly more differentiated form of stem cell, but ST-HSCs remain multipotent in their ability to support either lymphopoiesis (NK-cells, B-cells, T-cells) or myelopoiesis (erythrocytes, granulocytes, monocytes platelets) (Sender and Milo, 2021). The possibility that HSCs can also be segregated into distinct subpopulations that are biased from the outset towards a lymphoid or myeloid cell fate has been raised and attributed to differences in *Slamf1* (signaling lymphocytic activation molecule family member 1) expression levels in different HSCs. Specifically, those HSCs with high *Slamf1* express higher levels of myeloid specification genes whereas those with low *Slamf1* express higher levels of lymphoid specification genes (Beerman et al., 2010).

Aging in mouse models and in humans is associated with an increase in overall HSC numbers in the bone marrow (de Haan et al., 1997; Rossi et al., 2005; Geiger et al., 2013). This appears somewhat paradoxical given that aging is marked by the emergence of a spectrum of health issues tied directly to HSC dysfunction, including anemia, impairments in adaptive immunity, and increased blood-borne cancers (Jaiswal et al., 2014; Brosh et al., 2017; Bowman et al., 2018; Shlush, 2018; Groarke and Young, 2019; Jaiswal and Ebert, 2019; Lee et al., 2019; Lu et al., 2021). When the HSC population is evaluated as a whole, one of the most prominent features of aging in this stem cell compartment is a skewing of HSC differentiation towards myelopoiesis (Kuranda et al., 2011; Pang et al., 2011; Pang et al., 2017). The corresponding reduction in HSC-mediated support of lymphopoiesis probably explains, at least in part, the increased incidence of anemia as well as the compromised immunity observed with advancing chronological age. The mechanisms responsible for myeloid lineage skewing in HSCs are not fully understood, but this outcome appears to arise from a combination of extrinsic and intrinsic factors. For example, heterochronic transplantation studies in mice have shown that HSCs from aged donors fail to return to a balanced production of both myeloid and lymphoid cells following engraftment into young recipients, although a re-setting of the transcriptional profile in the aged cells towards one resembling HSCs of young adult mice does occur following transplantation (Kuribayashi et al., 2021). It is also known that HSCs of aged animals exhibit a variety of intrinsic genetic and epigenetic changes, which may result from aging-related disruptions in bone marrow niche signaling to the HSCs, random mutational errors that arise in HSCs from repeated cell divisions during life, oxidative damage within HSCs that lead to accumulation of DNA double-strand breaks, or combinations of the above (Akunuru and Geiger, 2016; Latchney and Calvi, 2017; Li et al., 2020; Mejia-Ramirez and Florian, 2020; SanMiguel et al., 2020; Zhang et al., 2020). The resultant changes in gene expression alter HSC function in such a way that only certain clones in the population effectively adapt to, and survive, changes in the bone marrow niche during aging. The end-result is that fewer and fewer HSCs serve as ancestors for the vast numbers of blood cells produced on a near-constant basis, all of which will carry the altered genetic make-up of the HSC clones that gave rise to them.

The ensuing over-representation of mature blood cells arising from the differentiation of individual HSC clones as animals age is commonly referred to as clonal hematopoiesis (Bowman et al., 2018; Shlush, 2018; Jaiswal and Ebert, 2019). Interestingly, clonal hematopoiesis was once thought to be exclusively linked to blood-borne cancers; however, there now exists clear evidence of this phenomenon in healthy subjects as well (Jaiswal and Ebert, 2019). To this end, several studies have examined the frequency of somatic clones within the bloodstream as humans grow older. Before the age of 40, somatic clones are nearly undetectable, being found in less than 1% of individuals; however, this frequency increases dramatically as humans age (Jaiswal and Ebert, 2019). One study found that almost 20% of blood cells in older individuals carry somatic mutations that can be traced back to clonal hematopoiesis (Jaiswal et al., 2014). With sufficient evidence to support the occurrence of clonal hematopoiesis, considerable research has focused on understanding its role in the emergence and progression of disease states with age through in-depth gene expression studies. For example, mutations in several protooncogenes, including *BRAF* (B-Raf serine-threonine kinase), *NOTCH1* (Notch 1 receptor) and *SF3B1* (splicing factor 3b subunit 1), have been found in hematopoietic progenitor cells isolated from leukemia patients (Damm et al., 2014; Jaiswal et al., 2014). These data suggest that tracing of aging-related clonal hematopoiesis may prove useful as a marker for preleukemia. Another gene often found mutated in clonal hematopoiesis is *TET2* (Tet methylcytosine dioxygenase 2), which encodes an enzyme involved in DNA demethylation. Mutations in *TET2* have been linked to a wide variety of pathologies, including leukemia, lymphoma, skewed hematopoiesis, and aberrant immune response regulation (Jiang, 2020). Deficiency in this gene has also been associated with severe inflammation and an upregulation of proinflammatory cytokines in response to bacterial infection (Jaiswal and Ebert, 2019). Collectively, these observations point to non-neutral clonal selection in this adult stem cell compartment as a key driver of numerous aging-associated pathologies.

## Oogonial stem cells

**FIGURE 3:**
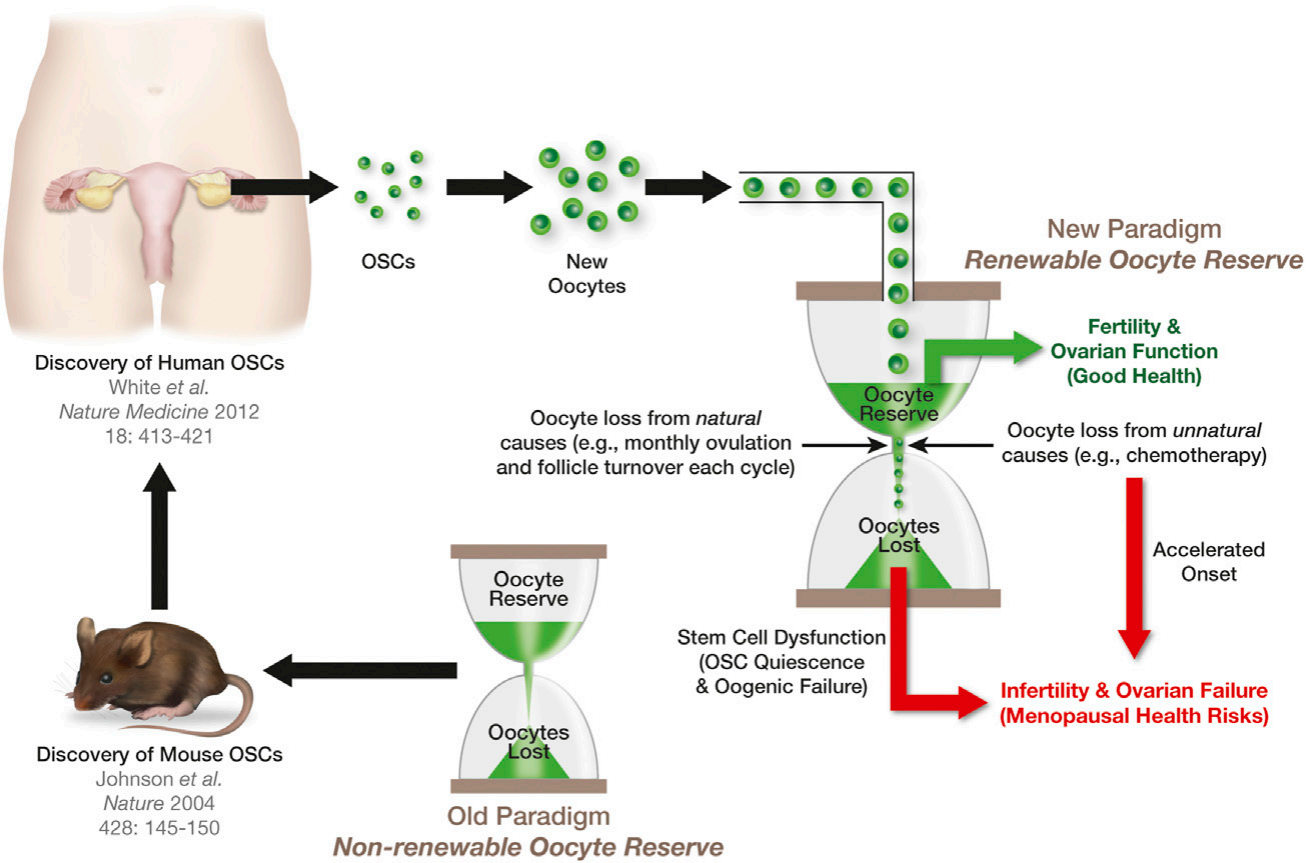
Significance of OSCs to ovarian aging and the menopause. The female biological clock can be viewed in the same manner as an hourglass, with each grain of sand in the upper reservoir representing a single oocyte in the ovaries. The loss of oocytes from this reserve naturally through atresia, or unnaturally from exposure to insults such as chemotherapy, is depicted as grains of sand running from the upper reservoir to the lower reservoir. The ovaries will function until such a time that the upper reservoir has run out of sand. The discovery of OSCs in mouse ovaries (Johnson et al., 2004) contradicted the long-standing paradigm that females are born with a nonrenewable oocyte population. The extension of this work to humans (White et al., 2012) raised the prospects that, in women, the upper reservoir of their biological clock is routinely replenished through stem cell-supported *de novo* oogenesis during adult life to sustain ovarian function (see also Alberico et al., 2022). However, the progressive quiescence of OSCs with age shuts down oocyte production, eventually leading to exhaustion of the oocyte reserve, ovarian failure, and the menopause.

While the causes and consequences of selfish clonal selection in SSCs with age have become more defined over the past several years, it is unknown if this same process occurs in the equivalent cell population in females. Like their male counterparts, OSCs are adult germline stem cells, the function of which is to support postnatal gametogenesis (*de novo* oocyte formation or oogenesis) in the ovaries (Figure 3). To date in mammals, OSCs have been identified in adult ovarian tissue of mice (Zou et al., 2009; White et al., 2012; Imudia et al., 2013; Park et al., 2013; Park and Tilly, 2015; Xiong et al., 2015; Zhang et al., 2016; Wang et al., 2017; Wu et al., 2017; Zou et al., 2019; Satirapod et al., 2020; MacDonald et al., 2021), rats (Zhou et al., 2014), pigs (Bai et al., 2013; Tsai et al., 2017; Hou et al., 2018; Nguyen et al., 2019), cows (de Souza et al., 2017), non-human primates (Woods and Tilly, 2015a; Li et al., 2021a), and humans (White et al., 2012; Woods and Tilly, 2013; Woods and Tilly, 2015b; Ding et al., 2016; Bothun et al., 2018; Clarkson et al., 2018; Silvestris et al., 2018; MacDonald et al., 2019; Ariyath et al., 2021; Sequeira et al., 2021; Alberico et al., 2022; Wu et al., 2022), and the cells have been isolated from ovaries of each of these species for detailed gene expression and functional characterization studies (reviewed in: Akahori et al., 2019; Martin et al., 2019). Once isolated and established in defined cultures, OSCs can be stably propagated long-term due to their inherent self-renewing capacity; additionally, OSCs are fully capable of completing meiotic differentiation into oocytes *in vivo* and *in vitro* (Martin et al., 2019). Also like their male counterparts, OSCs have been functionally defined through intragonadal transplantation-based approaches in mice and rats, which have consistently verified the ability of these adult stem cells to differentiate into new oocytes in recipient ovaries that mature, fertilize, and produce viable embryos and offspring (Zou et al., 2009; Zhang et al., 2011; White et al., 2012; Zhou et al., 2014; Xiong et al., 2015; Zhang and Wu, 2016; Wang et al., 2017; Wu et al., 2017). In addition, mouse OSCs incorporated into ovarian organoids maintained in defined cultures differentiate into oocytes that give rise to viable offspring following *in vitro* fertilization and embryo transfer (Li et al., 2021b). Genetic studies in mice, including inducible elimination of oocyte precursor cells through targeted suicide gene technology and inducible genetic lineage tracing, have further established both the importance of *de novo* oogenesis during adult life to ovarian lifespan as well as the physiological contribution of oocytes formed during adulthood to natural offspring production (Guo et al., 2016; Wang et al., 2017).

With respect to female reproductive aging, the discovery of mammalian OSCs nearly 20 years ago (Johnson et al., 2004) may initially appear at odds with the fact that the ovaries are the first major organ system to fail in females with age, which in humans leads to the menopause (Burger, 1996; Buckler, 2005; Broekmans et al., 2009). Ovarian aging is usually characterized as a decline in both the quantity and the quality of oocytes over time, up until a point at which the oocyte-containing follicle pool is essentially exhausted (Younis, 2012). While this event, and the onset of menopause that ensues, typically occur around 51 years of age, the timing of ovarian failure can be affected by a wide variety of factors, including diet, fitness, genetics, autoimmunity, and exposure to insults such as chemotherapeutic drugs (Cramer and Xu, 1996; Tilly, 2001; Mahmoud et al., 2007). From a mechanistic perspective, past studies have shown that aging of the ovary is multifactorial, but without question this process involves alterations in pathways responsible for protecting cells from oxidative stress (Tatone and Amicarelli, 2013; Sasaki et al., 2019; Tesarik et al., 2021; Wang et al., 2021; Yang et al., 2021). For example, with advancing age ovarian follicular granulosa cells exhibit a decrease in DNA double-strand break (DSB) repair capacity, which is paralleled by a sharp rise in DSBs (Zhang et al., 2015b). This has been mechanistically tied, at least in part, to impaired function of the protein encoded by the *BRCA1* (breast cancer 1) gene (Titus et al., 2013). In addition to changes in *BRCA1* levels, reduced expression of many key players in the cellular response to oxidative stress, including *IDH1* (isocitrate dehydrogenase (NADP (+)) 1), *PRDX4* (peroxiredoxin 4), and *NDUFB10* (NADH: ubiquinone oxidoreductase subunit B10), is a prominent feature of ovarian aging (Pizarro et al., 2020; Wang et al., 2020). In turn, the increase in oxidative stress and DNA damage heightens the propensity for granulosa cells to undergo apoptosis (Tilly and Tilly, 1995; Meng et al., 2020), which is central to the atretic degeneration of follicles in the ovaries and, consequently, the overall decline in oocyte numbers with age (Tilly, 1996; Tilly, 2001). With respect to oocyte quality, chronic administration of oral antioxidants in female mice has been reported to prevent the aging-associated increase in oocyte abnormalities used to define poor quality eggs, including spindle malformations, chromosomal errors, and mitochondrial distribution defects (Tarín et al., 1996). In turn, induction of oxidative stress in oocytes increases meiotic spindle abnormalities and chromosomal misalignment (Zhang et al., 2006). Other studies with mice have demonstrated that dietary caloric restriction, which is known to extend lifespan by minimizing oxidative damage with age (Sohal et al., 1994; Barja, 2002), is highly effective in preventing spindle and chromosomal defects in eggs of aged females (Selesniemi et al., 2011) as well as in dramatically extending functional ovarian lifespan into advanced ages (Selesniemi et al., 2008).

Like PAEs in males, maternal age effects (MAEs) are considered a prominent feature of human female reproductive aging (Gaulden, 1992; Leader et al., 2018). In addition to an increased risk for miscarriage, premature birth and perinatal mortality, advanced maternal age is also associated with a variety of fetal chromosomal errors and congenital abnormalities (Jacobsson et al., 2004; Cleary-Goldman et al., 2005; Delbaere et al., 2007; Fuchs et al., 2018). Regarding cancer, a retrospective cohort study of over 200,000 deliveries did not identify an association of maternal age with general malignant morbidity in offspring followed up to 18 years of age; however, childhood leukemia was found to independently associate with advanced maternal age (Imterat et al., 2018). Could at least some of these MAEs be due to non-neutral clonal selection in OSCs as females age, leading to the production of eggs which are compromised in terms of their developmental potential post-fertilization? The answer to this question is, unfortunately, unknown at present. If, however, clonal OSC selection is involved in conveying MAEs, evidence of the following should be available. First and foremost, human OSCs must exist. To that end, at least seven research groups have independently isolated OSCs from adult human ovaries since the initial report on the existence of these cells in ovaries of reproductive-age women in 2012 (White et al., 2012; Woods and Tilly, 2013). Moreover, all seven groups have rigorously established the germline identity and oocyte-forming capacity of the isolated cells (White et al., 2012; Woods and Tilly, 2013; Ding et al., 2016; Bothun et al., 2018; Clarkson et al., 2018; Silvestris et al., 2018; MacDonald et al., 2019; Ariyath et al., 2021; Sequeira et al., 2021; Wu et al., 2022). Second, OSCs should be, through the support of *de novo* oogenesis, active contributors to the pool of oocytes present in adult ovaries during reproductive life. This has been demonstrated in mice by *in-situ* mapping studies of active meiotic differentiation of endogenous germ cells in adult ovaries, genetic lineage tracing of endogenous germ cell developmental fate in adult ovaries, and evaluation of changes in oocytes numbers following targeted, but reversible, disruption of the meiotic differentiation of OSCs into new oocytes throughout adulthood (Guo et al., 2016; Wang et al., 2017). Along these same lines, very recent data support the occurrence of active germ cell meiosis in adult human ovaries *in vivo* as well (Alberico et al., 2022). Third, oocytes formed in the ovaries during adult life should be available and used by females for production of offspring under physiological conditions. This has been demonstrated by inducible genetic lineage tracing of germ cell fate in adult mouse ovaries followed by offspring mapping (Wang et al., 2017). Lastly, OSCs are, in a general sense, no different than other adult stem cell types in the body. Thus, it seems illogical that the same principles of niche deterioration with age and non-neutral clonal selection that are observed with somatic stem cells (HSCs) as well as with germline stem cells in males (SSCs) would not apply to germline stem cells (OSCs) in females.

## Epigenetic silencing of OSC differentiation with age

**FIGURE 4:**
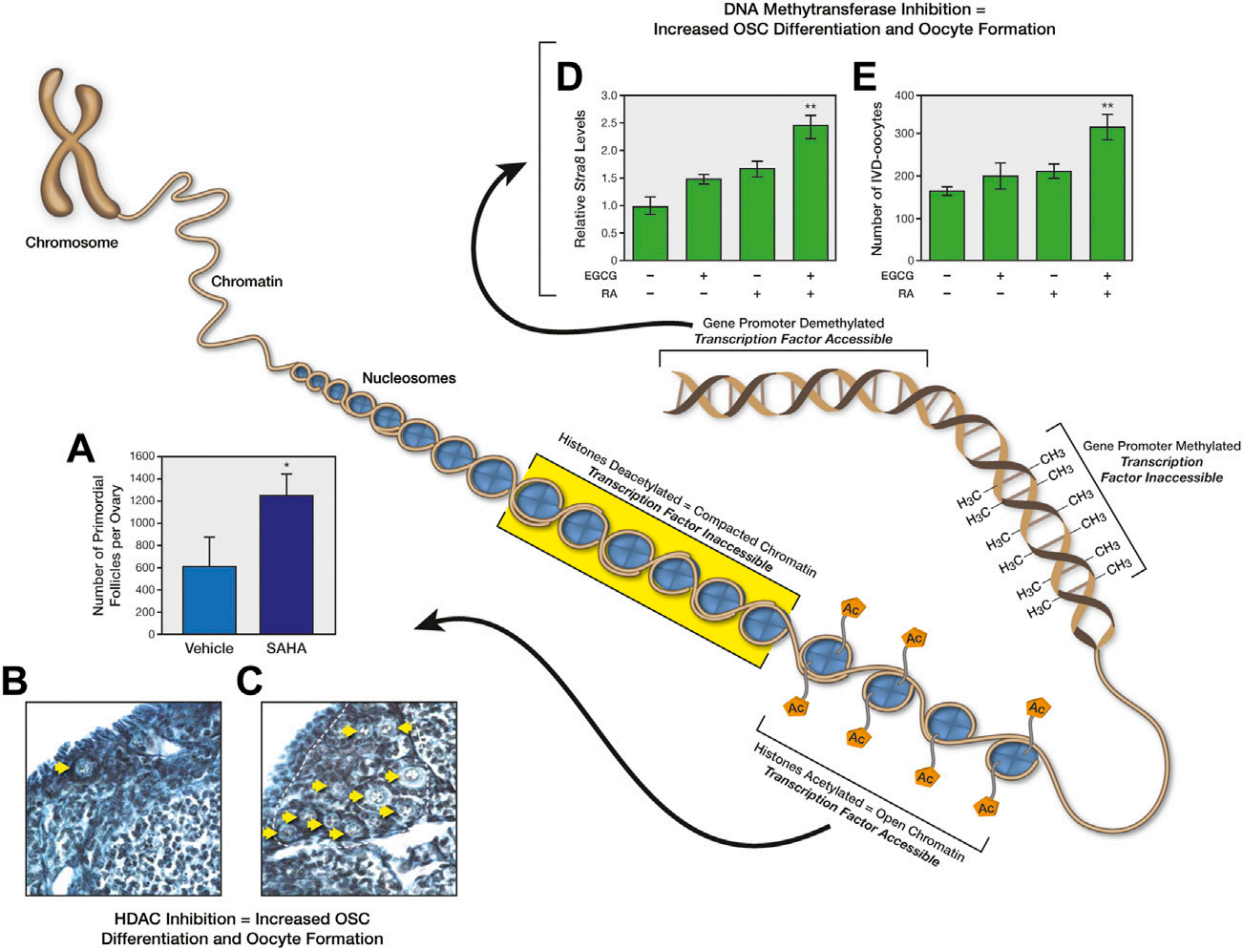
Epigenetic regulation of OSC differentiation and postnatal oogenesis. The diagram depicts how changes in chromatin structure, either through histone acetylation/deacetylation or DNA methylation/demethylation, controls accessibility of transcription factors to target genes, which in turn influences the differentiation of OSCs into oocytes. As one example, injection of reproductively aged female mice with the histone deacetylase inhibitor, suberoylanilide hydroxamic acid (SAHA), rapidly increases primordial oocyte-containing follicle numbers in ovaries **(A)**; representative ovarian sections of mice treated with vehicle **(B)** or SAHA **(C)** are shown for illustrative purposes, with yellow arrows highlighting the large increase in visible primordial follicles following SAHA treatment. These panels of data were reproduced from Wang and Tilly (2010). As another example, addition of the DNA (cytosine-5)-methyltransferase 1 inhibitor, epigallocatechin-3-gallate (EGCG), to cultures of mouse OSCs sensitizes the cells to retinoic acid (RA)-induced expression of the meiosis-commitment gene, *Stra8* (stimulated by retinoic acid gene 8) **(D)**, as well as to RA-promoted differentiation of the cells into in-vitro–derived (IVD) oocytes **(E)**. These panels of data were reproduced from Satirapod et al. (2020).

Although OSCs are active well into adulthood, there is clear evidence that the ability of these cells to maintain the reserve of oocytes *in vivo* diminishes as females grow older (Wang et al., 2017). Interestingly, however, the progressive quiescence of OSCs with age appears reversible, in that OSCs isolated from ovaries of postmenopausal women reactivate their oogenic potential when established as actively dividing germ cell cultures *in vitro* (Woods and Tilly, 2015b; Silvestris et al., 2018; Sequeira et al., 2021) or when transplanted into human ovarian cortical tissue for xenografting analysis (Wu et al., 2022). Similarly, OSCs isolated from female mice at very advanced ages can resume differentiation to form oocytes *in vitro*, and this correlates with the return of expression of a key epigenetic modulatory gene whose expression is lost in OSCs with age, *Dppa3* (developmental pluripotency-associated 3; also commonly referred to as *Stella*) (DC Woods and JL Tilly, unpublished observations). Past studies have established important roles for both nucleosomal histone modifications and DNA methylation in transcriptional activation of meiosis-related genes in germ cells and in the ability of OSCs to generate new oocytes (Wang and Tilly, 2010; Satirapod et al., 2020) (Figure 4). In addition, very recent data from studies of murine OSCs have shown that overexpression of *Dppa3* enhances their differentiation into oocytes, whereas targeted disruption of *Dppa3* through CRISPR/Cas9 promotes OSC proliferation at the expense of differentiation (Hou et al., 2022). These observations support that the loss of *Dppa3* expression in OSCs with age would lead to an ensuing decline in *de novo* oogenesis as a mechanism contributing to ultimate exhaustion of the oocyte-containing follicle reserve.

In view of these results, it would be interesting to test if reversible repression of critical genes like *Dppa3* in OSCs, through epigenetic modulation of chromatin conformation and transcription factor accessibility, represents a form of clonal selection in this stem cell lineage. Functionally, this would manifest as stem cell quiescence spreading across the population with advancing age. The end-result would be failure of the cells to differentiate and maintain regular oocyte production, leading to a cessation of ovarian function as the atretic depletion of follicles containing oocytes is no longer offset by oocyte renewal (Figure 2). Such a model aligns well with results from heterochronic transplantation experiments, which showed that *de novo* generation of oocytes and follicles in atrophic ovarian tissue collected from female mice at very advanced ages resumes when the tissue is removed and grafted into the ovarian bursal sacs of young adult female recipients (Niikura et al., 2009). Additionally, OSCs removed from aged human ovarian tissue can reactivate oogenesis *in vitro* (Woods and Tilly, 2015b; Silvestris et al., 2018; Sequeira et al., 2021; Wu et al., 2022). Thus, aging OSCs appear to at least retain the ability to mitigate one major aspect of ovarian aging–namely, to increase the *quantity* of oocyte-containing follicles. However, it remains unknown if the *quality* of oocytes produced by aging OSCs, which is the other key measure of ovarian aging, is compromised due to inherent genetic or epigenetic changes in the stem cells that arise with age and negatively impact on developmental potential of the eggs produced by those cells.

Returning to the question of whether non-neutral clonal selection in OSCs occurs in the ovaries with advancing age, it may be useful to consider what is currently known of clonal hematopoiesis and the mechanisms that contribute to this process. As discussed above, one of the primary drivers of mutations in HSCs, and the ensuing dysfunction in hematopoiesis with age, is believed to be elevated oxidative stress in the bone marrow niche (Lee et al., 2019). Oxidative damage is known to be a primary driver of single base substitutions (SBSs), such as SBS18 (Rouhani et al., 2016), across human tissues with age (Moore et al., 2021). A similar increase in oxidative stress has been observed in aging ovaries, which would increase the likelihood of random mutations accumulating within both somatic and germline ovarian cells (Sasaki et al., 2019; Tesarik et al., 2021; Wang et al., 2021; Yang et al., 2021). The task at hand is to identify those mutations which would be beneficial to continued function of OSCs with age but simultaneously be detrimental to the ability of oocytes derived from those cells to produce competent embryos for gestation and healthy offspring. Although the PAEs that arise from selfish selection in SSCs appear to involve primarily gain-of-function mutations that convey niche independence but disrupt normal patterning events in embryos conceived with sperm derived from the selected SSC clones, not all downstream outcomes may involve gain-of-function mutations in the stem cell population. For example, an inactivating mutation in a gene like *Txn1* (thioredoxin 1) would likely be tolerated by OSCs and oocytes, but the same mutation would be embryonic lethal if *Txn1*-null eggs derived from an OSC clone harboring that mutation were fertilized (Matsui et al., 1996). Such events might help explain the increased incidence of embryonic loss in women at advanced maternal ages, resembling to some degree what is seen with PAEs and selfish selection in SSCs. However, a larger issue that affects all women is the health risks associated with the onset of menopause resulting from aging-related ovarian failure, which is independent of fertility problems and MAEs. This is where the concept of progressive, but possibly reversible, stem cell quiescence with age could have the most bearing (Figure 3). Although more work is needed to test the possibility of clonal OSC selection in both female fertility as well as in the broader context of ovarian aging, numerous tools and models are now available which should facilitate this exciting line of study.

## Clonal selection vs. clonal dominance

Before closing, it is important for us to distinguish the concepts of non-neutral clonal selection discussed herein from the concept of clonal dominance. In stark contrast to the prospects of how clonal selection in stem cell populations can underlie the emergence of pathologies and tissue dysfunction with advancing age, clonal dominance refers to the process by which a small number of stem cells within a starting population give rise to a disproportionately large number of the descendent cells that ultimately contribute to a tissue during its formation or to the homeostatic maintenance of a tissue once it has been formed (Lopez-Garcia et al., 2010; Snippert et al., 2010; Gupta and Poss, 2012; Sereti et al., 2018; Alsous et al., 2021; Roan et al., 2021). Two of the most-well studied examples of clonal dominance in mammals are probably the sculpting of heart tissue during cardiac morphogenesis (Gupta and Poss, 2012) and the routine maintenance of intestinal crypt stem cells in the gut during adulthood (Lopez-Garcia et al., 2010; Snippert et al., 2010). Aside from the fact that non-neutral clonal selection has been functionally tied to the manifestation of disease states with age while clonal dominance plays a central role in normal tissue development and maintenance, clonal selection and clonal dominance differ in at least one other major respect–the mechanism by which each process likely occurs. In simplest terms, this mechanistic difference can be viewed in the context of whether competition among stem cell clones for survival and function in their niche is neutral or non-neutral (Stine and Matunis, 2013). In the case of neutral competition among stem cells of the same lineage, all stem cells in the population are genetically equivalent and possess an equal probability, over time, of being lost from the niche and replaced. In non-neutral competition among stem cells of the same lineage, genetic differences exist in individual clones of the population, and some of those differences promote the emergence of advantageous traits in certain clones over others for continued survival and function as animals age.

Referring back to the concept of non-neutral clonal selection in SSCs and HSCs, individual stem cells within the same population acquire random genetic or epigenetic changes, arising spontaneously from DNA replication errors or cumulative oxidative damage over time. Some of these changes enable certain clones to adapt, survive and function better under changing niche conditions that are generally disruptive to the self-renewal and/or differentiation capacity of the population as a whole. In the case of SSCs and spermatogenesis, this type of non-neutral clonal selection has been established as a major contributing factor to the increased risk of embryonic developmental abnormalities of paternal origin observed with advancing age (see Section 2. Selfish Selection in Spermatogonial Stem Cells). In HSCs, the same events are believed to underlie aging-associated lineage skewing favoring myelopoiesis over lymphopoiesis, and the spectrum of diseases that result from it (see Section 3. Lineage Skewing During Clonal Hematopoiesis). By comparison, clonal dominance does not rely on genetic or epigenetic heterogeneity developing in clones across the stem cell population due to mutational events occurring in individual cells but rather on a stochastic or neutral drift process involving a population of stem cells that is largely uniform in genotype and functional potency. In other words, clonal dominance occurs when a given stem cell is lost and then replaced by the symmetric multiplication of a neighboring stem cell, in the absence of either cell having a prior and inherent competitive advantage over the other. As this event is repeated over and over, clones within the population increase and decrease at random until a given clone either disappears or assumes a dominant role in generating many of the descendent cells in a tissue.

The occurrence of clonal dominance *in vivo* has been clearly illustrated by prior studies of intestinal stem cell crypt turnover in mice. By evaluating stem cell replacement vs. division rate in parallel with clonal fate mapping, Lopez-Garcia et al. (2010) demonstrated the importance of neutral drift and symmetrical stem cell divisions in adult stem cell homeostasis and maintenance of normal tissue function under physiological conditions (Lopez-Garcia et al., 2010). Likewise, studies of oogenesis in *Drosophila* have provided intriguing insights into potential mechanisms underlying the occurrence of clonal dominance during organogenesis. In female flies, oocytes develop within a series of egg chambers, each of which is comprised of a germline cyst enclosed by a unilaminar follicle epithelial cell layer. As egg chambers increase in size due to germline expansion, the number of cells comprising the follicle epithelium, which is initially derived from two follicle stem cells (FSCs) located in the stem cell niche of the ovariole, increases by approximately 20-fold (Margolis and Spradling, 1995; McLaughlin and Bratu, 2015). During this time, daughter cells that arise from the two different FSC clones undergo cell division but remain interconnected by ring canals, the latter of which facilitates reconstruction of lineage trees. Using fluorescence microscopy and lineage mapping, coupled with robust mathematical modeling, Alsous et al. (2021) recently showed that the majority of follicle epithelial cells surrounding the “oldest” (stage 6) egg chamber are derived from only one of the two FSCs that initially give rise to the epithelium surrounding the “youngest” (stage 1) egg chamber. Strikingly, this example of clonal dominance, which leads to expansion of the follicle epithelium, occurs spontaneously through the coupling of divisions among interconnected descendant cells.

## Conclusion and future directions

Over the past several years, a strong connection between stem cell mutations and a spectrum of health problems with age has emerged (Adams et al., 2015). Based on studies of non-neutral clonal selection in SSCs and HSCs discussed earlier, as well as recent evidence from other models (Sirvinskas et al., 2020), stem cells in a tissue cannot be viewed as a relatively homogeneous population of cells but rather as a heterogenous mix of cells comprised of individual clones that can vary significantly from each other in gene expression patterns and function. It is therefore imperative that future studies examine stem cells of various lineages individually, an approach that will be greatly facilitated by standardized protocols and workflow optimizations for single-cell genomics, single-cell RNA sequence analysis (scRNA-seq), and single-cell proteomics. As these and other analytical tools are eventually applied to assessment of clonal heterogeneity and non-neutral selection in adult stem cell populations other than SSCs and HSCs, the answer to the question of whether clonal selection with age is a fundamental principle that applies to all types of adult stem cells or only to a select few will be obtained.

This line of work will also address if gene mutation-based clonal selection in stem cell populations involves genetic alterations that are somehow advantageous to a select group of clones but ultimately disrupt the downstream function of the progeny derived from those cells. For SSCs, the concept of selfish selection has been well established. However, this type of clonal selection may not universally apply to all adult stem cell lineages. For HSCs, clonal hematopoiesis with age clearly occurs, but this process appears to primarily revolve around unbalanced differentiation of HSCs toward one lineage rather than specific gene mutations that enable certain HSC clones to thrive independent of aberrant signaling from the aging bone marrow niche at the expense of downstream hematopoietic function, broadly defined. Although some pathologies associated with aging of HSCs can be traced back to alterations in specific genes and age-related expansion of clones containing those mutations, to our knowledge there is currently no direct evidence for selfish selection in HSCs. Thus, while non-neutral clonal selection with age may occur differently depending on the stem cell lineage, the end-result may be the same–aberrant function of the tissue normally supported by that population of stem cells. In the case of OSCs, time and experiments will tell if clonal selection occurs in this stem cell lineage. It is known that OSCs persist in the female gonads long past the time of aging-related ovarian failure, but the cells no longer support *de novo* oocyte production. As discussed earlier, this change in function appears reversible in that aged OSCs can resume oogenesis under certain experimental conditions. Hence, it may be that quiescence of this stem cell lineage with age is how clonal selection manifests, and this may reflect epigenetic changes in the cells which progressively impair their differentiation potential.

This leaves one other major question to be considered. Do stem cells maintained in culture undergo their own form of advantageous clonal selection, or “evolution”, in response to suboptimal environmental (culture) conditions that are far from what would normally be experienced by those cells in their niche(s) *in vivo*? To this end, a recent study of induced pluripotent stem cells found that sub-clonal diversity of an initially heterogeneous population of stem cells declined markedly as the cells were passaged multiple times (Brenière-Letuffe et al., 2018). These observations support that some cells within the initial stem cell population were better adapted than others to survive and thrive under the specific culture conditions applied to that population. If clonal selection is shown to be a consistent “artifact” of culturing stem cells, this would complicate the interpretation of results from published studies which approached the population as a homogenous pool or as being reflective of the population *in vivo*. However, this artifact of stem cell culture could also offer great opportunity, since it would provide a tightly controlled experimental system to rapidly elucidate the genes and pathways selected for in those clones that survive, and thrive, *in vitro* when compared to the starting population of cells prior to their establishment in culture. By evaluating these outcomes in context with what is known of the endogenous niche-derived cues that normally support self-renewal and/or differentiation of the stem cell lineage in question, targets or leads may emerge that would enable further testing of the dynamics of that stem cell population *in vivo*. Using these experimental paradigms and techniques, we are currently exploring the possibility that clonal selection in OSCs, like that in HSCs and SSCs, not only occurs during adult life but also plays a key contributory role in ovarian aging and, possibly, the emergence of MAEs. This information will also be critical for optimization of emerging stem cell-based research platforms aimed at reconstitution of oogenesis in ovarian tissue for various fertility applications (Akahori et al., 2019; Li et al., 2021b).
